# The importance of parameter choice in modelling dynamics of the eye lens

**DOI:** 10.1038/s41598-017-16854-9

**Published:** 2017-11-30

**Authors:** Kehao Wang, Demetrios T. Venetsanos, Jian Wang, Andy T. Augousti, Barbara K. Pierscionek

**Affiliations:** 1 Faculty of Science Engineering and Computing Penrhyn Road Kingston-upon, Thames, KT1 2EE UK; 2 College of Science and Technology Nottingham Trent University, DH Lawrence Building, Clifton Campus Clifton Lane, Nottingham, NG11 8NS UK

## Abstract

The lens provides refractive power to the eye and is capable of altering ocular focus in response to visual demand. This capacity diminishes with age. Current biomedical technologies, which seek to design an implant lens capable of replicating the function of the biological lens, are unable as yet to provide such an implant with the requisite optical quality or ability to change the focussing power of the eye. This is because the mechanism of altering focus, termed accommodation, is not fully understood and seemingly conflicting theories require experimental support which is difficult to obtain from the living eye. This investigation presents finite element models of the eye lens based on data from human lenses aged 16 and 35 years that consider the influence of various modelling parameters, including material properties, a wide range of angles of force application and capsular thickness. Results from axisymmetric models show that the anterior and posterior zonules may have a greater impact on shape change than the equatorial zonule and that choice of capsular thickness values can influence the results from modelled simulations.

## Introduction

The eye lens is a lamellar structure of fibre cells^1^ that accrue throughout life on the surface forming layers over existing tissue with no concomitant cellular loss^2^. A basement membrane called the capsule^2^ encases the lens and the organisation of proteins and water within the fibre cells preserves the requisite degree of transparency throughout a greater part of life. The lens contributes a third of the refractive power of the eye and it is solely responsible for fine-tuning focusing so that objects over a wide range of distances can be seen clearly^1,2^. The process of altering the focussing power of the eye, termed accommodation, is activated by the ciliary muscle, which mediates forces to the capsule through a ring of fibrous ligaments collectively called the zonule. The zonule is broadly divided into anterior, equatorial and posterior sections^3^ that insert into the capsule around the equatorial zone. With age there is a decline in accommodative amplitude^4,5^: a process that eventually leads to a refractive condition called presbyopia. This manifests in a loss of capacity to focus on near objects^6,7^ by the fifth to sixth decades of life.

Theories that explain accommodation and its loss remain controversial^8^, and relate to how the zonular forces are exerted on the lens and how the lens surface curvatures change during the act of accommodation^9–12^. A comprehensive and definitive understanding of the mechanism of accommodation is needed, as are age-related changes to lens mechanical properties^13^, in order to progress the design of intraocular implant lenses to mimic the eye lens in both structure and functional capacity. To date, no implant lens can provide the optical quality or accommodation that is given by the biological lens. It has been widely agreed that the lens becomes more resistant, with age, to external deforming forces^14,15^ resulting from changes in elastic moduli^16–18^. In addition, the reduced capsular elasticity^12,19^, the change of the relative positions of zonular attachments on the capsule^7,20,21^ and the decreased zonular tension due to the continued growth of the lens have all been postulated to contribute to presbyopia.

The equatorial zone of the lens, which is the site of ciliary muscle force transmission via the zonule to the lens and capsule has been visualized *in vivo* ^22–24^. However, definitive questions about the mechanism of accommodation remain unanswered. Finite Element Analysis (FEA), in combination with *in-vivo* and *in-vitro* studies, can provide insights into functional changes of the lenses during accommodation^25–29^ as well as evaluating surgical effects^30,31^. Many recent studies have used this computational method to model lens behaviour with simulated radial stretching that mimics accommodation^27,32,33^. The zonular configurations have been given comparatively little attention in modelling studies although the magnitude and direction of stretching forces had been considered^27^. One recent investigation has considered this important aspect of accommodation and demonstrated the effect of zonular forces on the curvature of the anterior and posterior lens surfaces^25^. Greater insights into the effect of the zonular angles notably the directions of the zonular forces, on the accommodated state of the human lens are required.

Effective implementation of FEA and creation of physiologically relevant models relies greatly on the accuracy of experimental values of material properties as well as on the geometry of the lens and zonule. Various approaches have been considered in previous work; the lack of consistency in values and age-related trends can be explained by the range of diverse methods and variations in the measured locations within lenses^16,18,34–37^. Studies using spinning^16,17^ and indentation methods^34^ have reported stiffer lens cortices than nuclei in younger ages and the stiffness in both parts increases with age at different speeds. Brillouin scattering analysis performed on living lenses^37^, demonstrated a stiffer nucleus than cortex with little dependence on age^36,37^.

This work presents the results of an *in-silico* parametric study conducted on a range of different axisymmetric models and shows the importance of correlations between a range of combinations of zonular angles, as well as the effect of these angles on the performance of lens models. The study further investigates two different sets of material properties reported in the literature from studies that have measured elastic moduli of intact eye lenses using centrifugal forces^16,17^ and compares the optical performance provided by models with uniform^19,38^ and spatially varying capsular thicknesses^39^.

## Results

**Figure 1:**
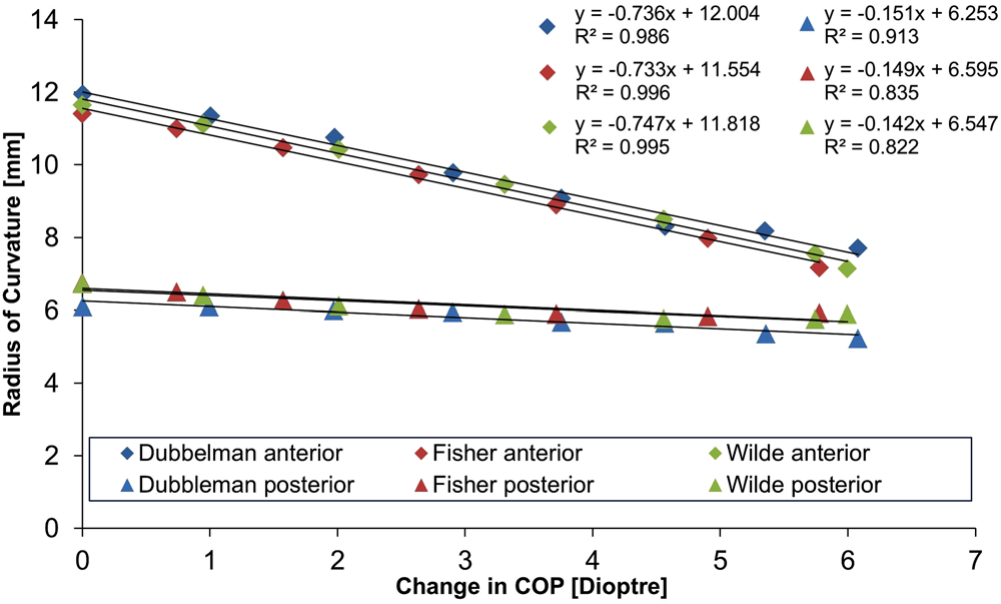
Radius of curvature in mm plotted against the change in COP in dioptres (D) showing the optimal combinations of zonular angles that fit most closely to *in-vivo* data^40^ for the 16-year-old lens model using both sets of material properties^16,17^ when applied with a displacement set of 0.5 mm to the equatorial zonule, 0.6 mm to the anterior and posterior zonule.

The changes in radius of curvature of both anterior and posterior surfaces of lens models plotted against the change in Central Optical Powers (COP) were compared with the changes reported in an *in-vivo* lens^40^. Displacements applied to the zonular sections of 0.5 and 0.6 mm were based on values of maximal changes in ciliary ring diameter with accommodation that were shown to range from 1.0 mm to 1.2 mm^41^. Combinations of 0.5 and 0.6 mm displacements were tested and optimal fits to *in-vivo* data found for 0.5 mm displacement applied to the equatorial zonule and 0.6 mm displacements applied to the anterior and posterior zonular fibres. This is shown in Fig. 1 for the models of the 16 year old lens with a uniform capsular thickness of 13 μm^19^ using both the material properties of Fisher^16^ and Wilde *et al*.^17^.

**Figure 2:**
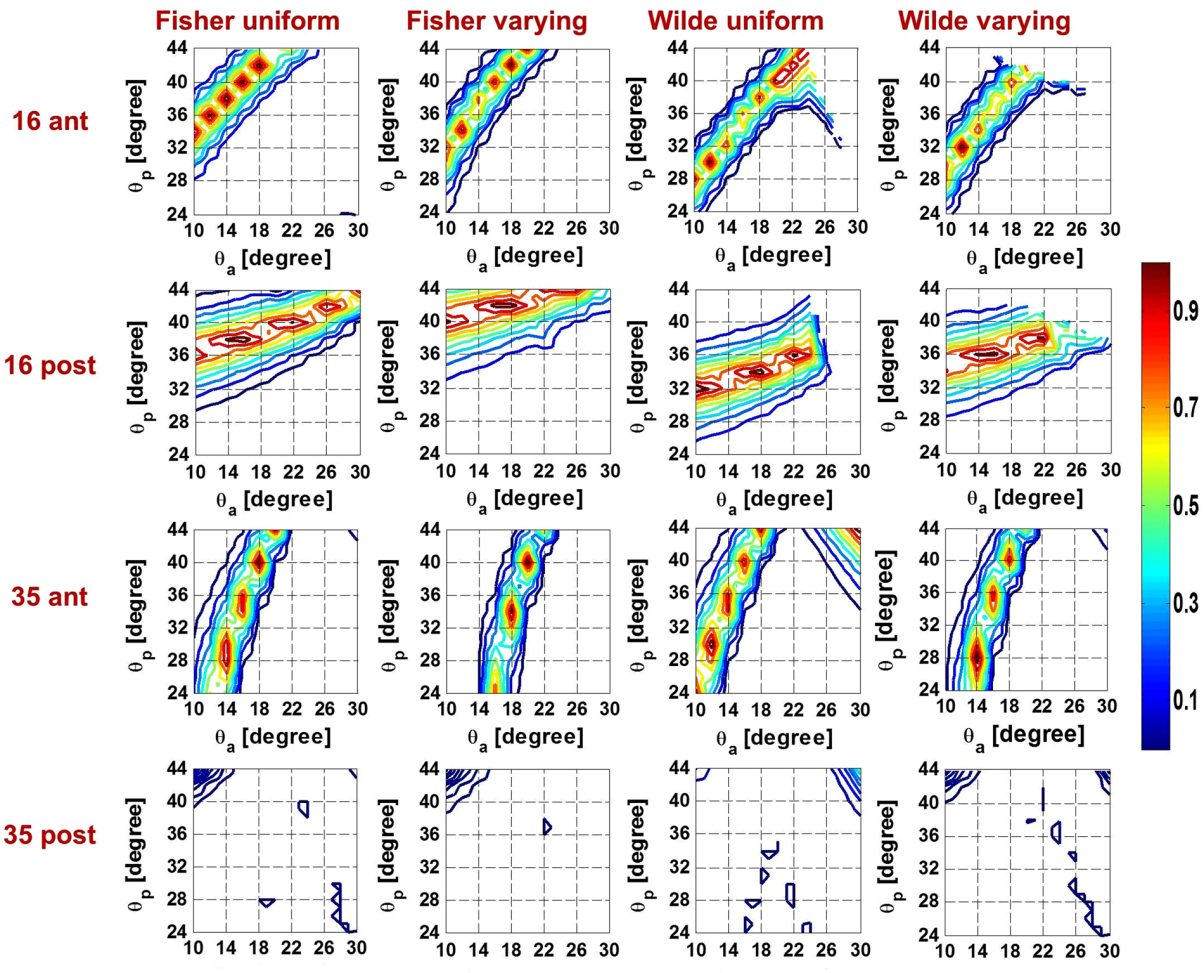
The p-value contours showing relations between anterior and posterior zonular angles when the equatorial zonular angle was fixed to 2 degrees for both the anterior and posterior lens surface of models aged 16 and 35 years old two sets of using different material properties and capsular thickness.

The relative contributions of the anterior zonular angle, ${\theta }_{a}$, equatorial zonular angle, ${\theta }_{e}$, and posterior zonular angle, ${\theta }_{p}$, on accommodation were investigated by fixing ${\theta }_{e}$ at 2° and plotting contours of the p-values corresponding to ${\theta }_{a}$ and ${\theta }_{p}$ in Fig. 2 for both anterior and posterior surfaces of models with various modelling parameters. The magnitude of the p-values is represented by the contour colours with the colour bar shown on the right hand side; p-values that are close to 1, and which indicate the best match of model to *in-vivo* data^40^, are represented by contours shown in red. As the colours move from red to blue the p-values decrease, as shown by the scale bar and there is less likelihood of a match to *in-vivo* data (Fig. 2).

When using material properties from Fisher^16^, the bands of p-values for the 16-year-old lens model are relatively distinct and this is particularly so for the anterior surface. The contours are slightly less crisp for the anterior surface of the 35-year-old model than those seen for the anterior surface for the 16-year-old model with material properties of Fisher^16^ but are nevertheless relatively well defined. There are no clear bands of p-values for the posterior surface of the 35-year-old model. The posterior p-value contours are only seen in dark blue (the lower range of p-values) and are mainly concentrated where ${\theta }_{p}$> 38° and ${\theta }_{a}$ < 16°.

When using material properties of Wilde *et al*.^17^, there is less distinction in the p-value range for the 16-year-old model. The 35-year-old model is similar to the one using material properties of Fisher^16^ in that the anterior lens surface demonstrates a substantially improved fit to *in-vivo* data^40^ when compared to the posterior surface and the maximal p-value for the anterior surface almost ten times greater than its counterpart for the posterior surface. Some additional bands of p-values seen for higher ${\theta }_{a}$ and ${\theta }_{p}$ with the 35-year-old model using material properties of Wilde *et al*.^17^ compared with the model using the material properties of Fisher^16^. For this age model, there is no angle combination within the tested ranges that could be found to fit both surfaces using either set of material properties, and this is evidenced by the lack of overlapping contour regions in 3^rd^ and 4^th^ row of Fig. 2.

The equatorial zonular angle ${\theta }_{e}$ set for angles of −14°, −8°, −2°, 2°, 8° and 14° is shown in supplementary figures 1–4. Supplementary figures 1 and 2 give results for models using material properties of Fisher^16^ with a uniform^19^ and spatially varying capsular thicknesses^39^ respectively; supplementary Figures 3 and 4 show the counterpart models using material properties of Wilde *et al*.^17^. There is little distinction between patterns of p-values between uniform and varying capsules in all but the 16-year-old model with the material properties of Wilde *et al*.^17^ for which, in the varying capsular thickness model (supplementary Figure 4a and b) the more defined p-value bands, that would indicate a better fit with *in vivo* data, span a greater range of ${\theta }_{a}$ and ${\theta }_{p}$ than found in the corresponding model with uniform capsular thickness (supplementary Figure 3). This is particularly notable for the anterior surface (supplementary Figures 3a and 4a). For both surfaces of the 35-year-old model using two sets of material properties^16,17^ and for the 16-year-old model using material properties of Fisher^16^, there is little change with varying ${\theta }_{e}$ indicating that the angle of the equatorial zonule makes a negligible difference. For the 16-year-old model using material properties of Wilde *et al*.^17^, there is greater variation as ${\theta }_{e}$ is changed.

The zonular angle triplets that provide the optimal fits for anterior and posterior surfaces for models using both material properties of Fisher^16^ and Wilde *et al*.^17^ are shown in Table 1 (upper section). These only refer to 16-year-old models as no 35-year-old model with sufficiently adequate fits to *in-vivo* data^40^ for the posterior surface could be found. For the model with a varying capsular thickness and material properties from Fisher^16^ the optimal fit to *in-vivo* data is for ${\theta }_{a}$ = 18°, in the remaining three cases the optimal fit is for ${\theta }_{a}$ = 14°. There is less consistency in ${\theta }_{p}$ the optima of which vary between 34° to 42°. Both anterior and posterior p-values are higher for models with material properties from Fisher^16^ than for those from Wilde *et al*.^17^; the change in COP are similar.

**Table 1:** Zonular angle triplets resulting in the optimal fittings to *in-vivo* data^40^ for 16-year-old models and zonular angle triplets resulting in the maximal change in COP for both aged models.

Optimal fits					
		(***θa***, ***θe***, ***θp***)	Anterior p-value	Posterior p-value	Change in COP
Fisher^16^	16 uniform	14°, −6°, 38°	0.942	0.939	5.8
	16 varying	18°, 6°, 42°	0.962	0.956	5.9
Wilde^17^	16 uniform	14°, 14°, 34°	0.810	0.801	6.0
	16 varying	14°, 14°, 36°	0.730	0.937	6.0
**Maximal change in COP**					
		**Capsular thickness [µm]**	**(** ***θa***, ***θp*** **)**	***θe***	**Change in COP [dioptre]**
Fisher^16^	16 uniform	13	10°, 24°	[−14°, 14°]	8.7
		20			7.3
		5			11.0
	16 varying	—			10.6
	35 uniform	15			5.8
		20			5.0
		7			7.8
	35 varying	—			7.2
Wilde^17^	16 uniform	13	10°, 24°	[−14°, 14°]	8.8
		20			7.5
		5			9.7
	16 varying	—			9.6
	35 uniform	15			5.1
		20			4.4
		7			6.8
	35 varying	—			6.3

In order to test the effect of varying the values of uniform capsular thickness on the maximum change in COP, three uniform capsular thickness values incorporating ranges reported in the literature^19,38^ were modelled and shown with the corresponding change in COP in the lower part of Table 1. This is calculated for all tested models (ie for both aged models with different configurations of capsular thickness using both sets of material properties). The resulting maximal change in COP do not vary with ${\theta }_{e}$ within the range of [−14°, 14°]. The results show that for the same levels of displacement, varying capsular thickness models produce a greater change in COP than uniform capsular thickness models when the thickness of 13 µm^19^ or 20 µm^38^ (values found for the anterior capsule) but a slightly lower change in COP than models with a uniform capsular thickness of 5 µm^38^ (value measured for the posterior lens capsule). The 16-year-old lens models demonstrated a greater change in COP than the 35-year-old lens models. It is notable that the zonular angle triplets that produce the highest change in COP are different from those that provided the optimal fits to the *in-vivo* lens^40^.

**Figure 3:**
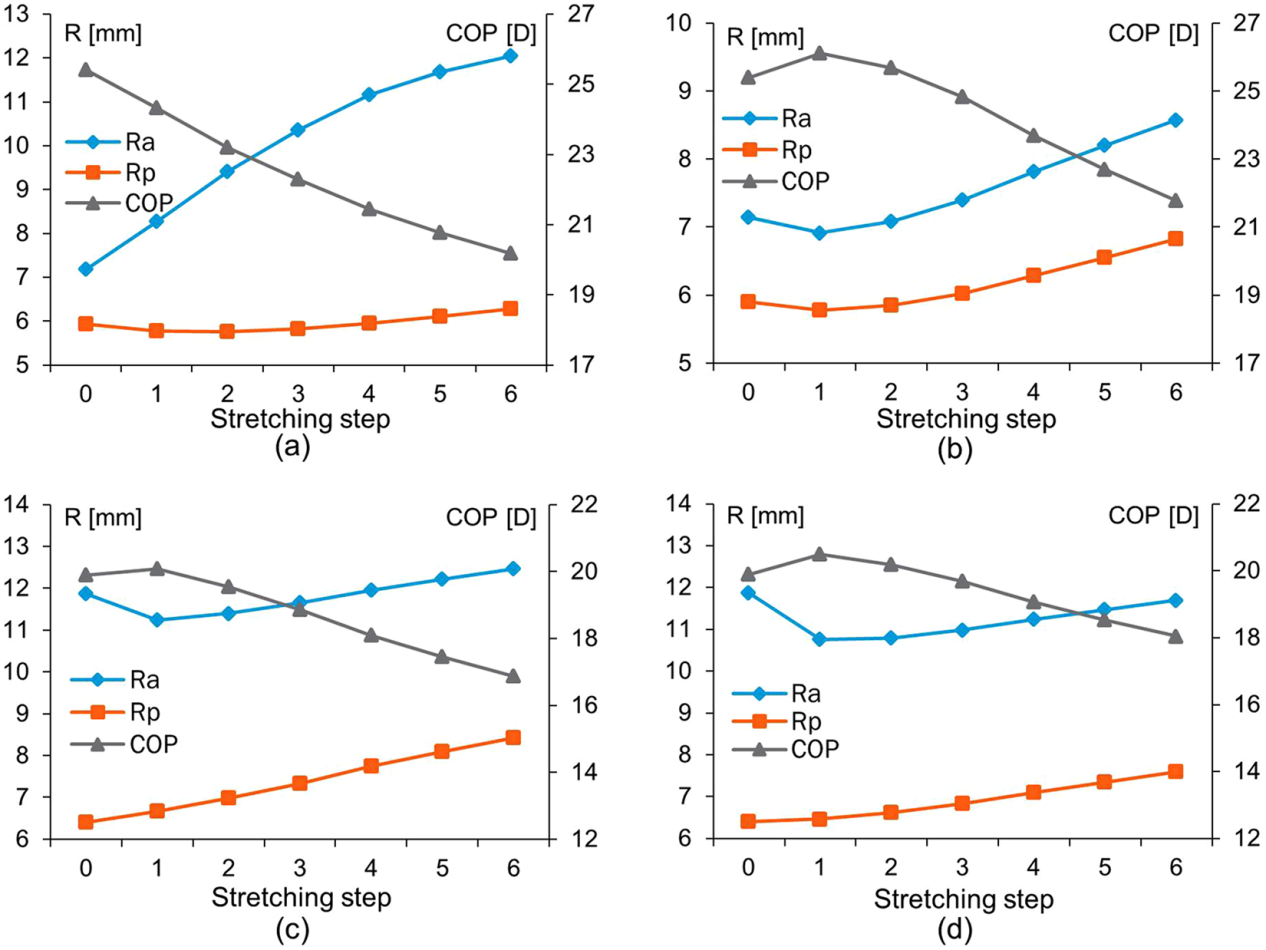
The changes in radii of curvature (R) in mm and Central Optical Power (COP) in dioptres (D) for progressive stretching steps of the 16-year-old lens model using material properties of (**a**) Fisher^16^ and of (**b**) Wilde *et al*.^17^ and of 35-year-old lens model using material properties of (**c**) Fisher^16^ and of (**d**) Wilde *et al*.^17^.

Figure 3 shows changes in radii of curvature of the anterior and posterior lens surfaces and in Central Optical Power (COP) (ie the power along the optic axis) for progressive increments of simulated stretching step for the 16 and 35 year old models with the two sets of material properties^16,17^ and a uniform capsular thickness^19^. The selected zonular angle triplet for each model demonstrated nonlinear behaviour that did not compare with the *in-vivo* data. In Fig. 3a, which shows the 16-year-old lens with material properties from Fisher^16^, the COP decreases steadily as ${r}_{a}$ increases with very little change in ${r}_{p}$. For the 16-year-old lens model using the material properties of Wilde *et al*.^17^ (Fig. 3b) and the 35-year-old lens models using both sets of material properties^16,17^ (Fig. 3c and d), the increase in ${r}_{a}$ is not immediate with the first stretching increment but rather show a slight decrease then followed by an increase in radius but along a shallower slope than seen in Fig. 3a; the COP behaves in a reciprocal way (Fig. 3b,c and d). There is a greater rate of increase with stretching in ${r}_{p}$ for these models (Fig. 3b,c and d) than for that seen in Fig. 3a.

**Figure 4:**
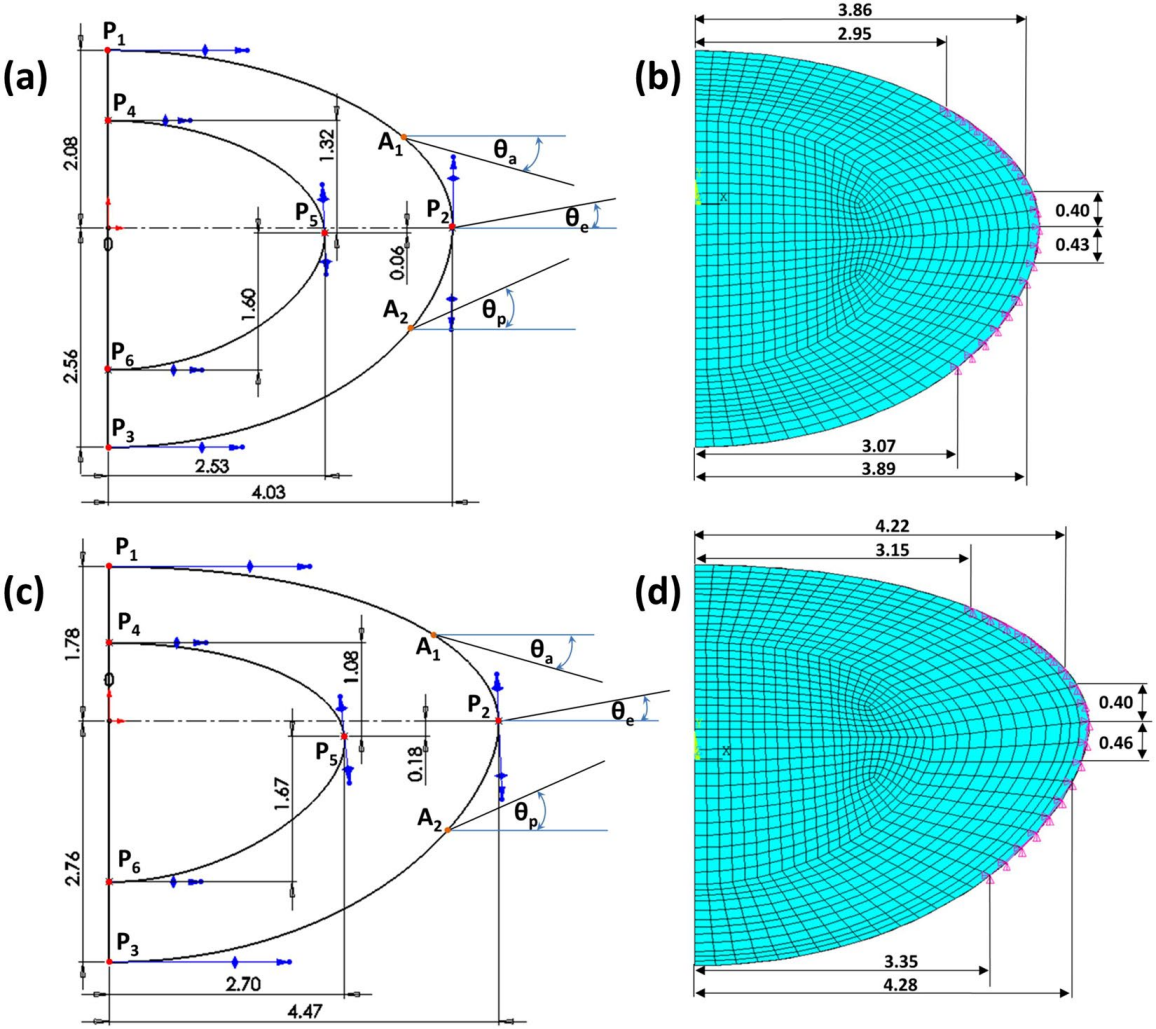
The geometries and dimensions of the (**a**) 16-year-old lens and (**c**) 35-year-old lens as well as the anchorage regions of zonular fibres on lens capsule for the (**b**) 16-year-old lens and (**d**) 35-year-old lens.

## Discussion

Models offer a perspective that is not currently possible from *in-vivo* studies. The models described in this study were based on geometries measured on *post-mortem* lenses freed from zonular attachments^42^ and were compared to results from *in-vivo* data^40^. The results of this study indicate the importance of choice of material properties and force directions ie zonular angles in creation of physiologically relevant lens models if these are to be used to predict accommodative behaviour for a range of applications such as design of accommodating implant lenses that mimic the action of the biological lens. In particular this work shows that the anterior and posterior zonules have a greater effect on lens shape change than the equatorial section of the zonule and that the quality of the fits to *in-vivo* data varies substantially with different zonular angle triplets (Fig. 2). The values of the anterior and posterior zonular angles both have specific ranges for which good fits were found (Fig. 2). The important roles in altering the anterior and posterior lens curvatures played by the anterior and posterior zonules were demonstrated in an experimental stretching study^43^. The optical performance of lens models, which is ultimately the pertinent parameter for the eye, are also affected by the zonular angles as the changes in COP vary with different zonular angle triplets (Table 1).

Given the breadth of individual variations that can mask ageing trends^44^, in addition to differences between the *in-vivo* lens and the *in-vitro* lens, the models in this study were not intended to represent the behaviour of all lenses at the respective modelled ages. Rather, they represent more general trends and show how accommodation can change depending on geometry and material properties; whilst ageing is a factor that affects shape and material parameters, individuals manifest ageing at different rates.

When considering the fits of both surfaces simultaneously, only the 16-year-old lens model provided both p-values above the significance level for certain zonular angle triplets (Fig. 2). A 48-year-old lens model, considered in previous work^25^ provided a significantly lower change in COP, for the same amount of displacement, than found with younger lens models aged 16 and 35 years. This is consistent with the fact that lenses in the fifth decade have reduced capacity to accommodate.

Anatomical studies using scanning electron microscopy have revealed that the majority of the anterior and posterior zonular fibres originate from the pars plana of the ciliary body and link forward to the posterior pars plicata where they are fixed to the ciliary processes through small strands of fibre bundles^45^, from which they split into two strands connecting respectively to the anterior and posterior lens capsule^45,46^. The equatorial fibres, mainly originating from the pars plicata, are sparse and poorly developed compared with the anterior and posterior fibres^47^. The angles of the anterior and posterior zonular sections that exert tension on the lens are controlled by the morphology of ciliary body and their relative location to the lens equator. The increase in lens dimensions^41,48,49^, the decrease in the inner ciliary body diameter^44,50,51^ as well as the anterior movement of the zonular insertions^20^ with age could all contribute to a shift in zonular angles resulting in zonular forces becoming more tangential to the lens surface with age and therefore less able to exert tension on the lens capsule^52,53^. The zonular angle triplet resulting in the maximal change in COP occurs for an anterior angle of 10° and a posterior zonular angle of 24°, which is different from the values that provide the optimal fits to *in-vivo* data^40^ (Table 1). This difference demonstrates the changing zonular angles with age could contribute to the decline of lens accommodative ability.

The capsular thickness varies along its length and changes with age^39,54^. In general, the thickness of the anterior portion is five to ten times thicker than the posterior portion^38,39,55^ and the effect of age on capsular thickness is greater for the anterior than the posterior section^54,55^. The capsule of an accommodating lens is thickest in the regions that coincide with the anterior and posterior zonular insertions^12,39,54^. The strain energy stored within the capsule during deformation is directly related to the capsular thickness^38,56^ and is therefore considered able to influence lens shape change during accommodation. The 16-year-old model using the material properties of Wilde *et al*.^17^ appears to be more sensitive to capsular thickness variations with more clearly defined regions of p-value bands (Supplementary figure 4) than for the corresponding uniform capsular thickness model (Supplementary figure 3). This may be because the moduli reported by Wilde *et al*.^17^ for a lens of this age are significantly lower than those reported by Fisher^16^ (Table 2), indicating a more pliable lens that could be more sensitive to alterations in capsular thickness.

**Table 2:** Shape parameters and material properties.

Geometrical parameters						
Age	Point	x coordinate[mm]	y coordinate[mm]	Tangent weighting 1	Tangent weighting 2	Tangent radial direction [degree]
16	P_1_	0.00	2.08	0.00	6.75	0.000
	P_2_	4.03	0.00	3.16	4.24	−90.77
	P_3_	0.00	−2.56	6.51	0.00	180.00
	P_4_	0.00	1.26	0.00	3.98	0.00
	P_5_	2.53	−0.06	2.13	1.84	−87.31
	P_6_	0.00	−1.66	4.56	0.00	180.00
	A_1_	3.46	1.04	—	—	—
	A_2_	3.54	−1.19	—	—	—
35	P_1_	0.00	1.78	0.00	9.61	0.00
	P_2_	4.47	0.00	2.23	3.37	−87.44
	P_3_	0.00	−2.76	8.60	0.00	180.00
	P_4_	0.00	0.90	0.00	4.62	0.00
	P_5_	2.70	−0.18	1.77	1.98	−83.74
	P_6_	0.00	−1.84	4.40	0.00	180.00
	A_1_	3.73	0.99	—	—	—
	A_2_	3.88	−1.26	—	—	—
**Material properties**						
	**Fisher** ^16^ **in Young’s modulus [kPa]**			**Wilde** ^17^ **in shear modulus [kPa]**		
	**nucleus**		**cortex**	**nucleus**		**cortex**
16	0.50		2.40	0.06		0.32
35	0.60		3.70	0.26		0.89

The change in COP seen in lens models with varying capsular thicknesses were higher (for the same levels of force) than those from the uniform capsular thickness models with thickness values taken from anterior lens surfaces^19,38^, but were slightly lower than those from the models with uniform capsular thickness values taken from the posterior lens capsule^38^, for both ages using both sets of material properties (Table 1). It is not definitively known whether the varying capsular thickness is a consequence of stress-induced modelling of the capsule^38^, whether it serves a functional purpose in contributing to changes in the curvature of the accommodated lens^12,19,56,57^ or whether it acts only as a distributor of force^58^. The present study demonstrated the important influence of capsular thickness in determining the optical performance of lens models as the change in COP varies depending on the choice of capsular thickness, whether uniform or varying.

Modelling can offer perspectives on accommodation to test theories and provide insights into how models with different parameters align with theoretical predictions. The current theories of accommodation which are regarded as opposing, propose different explanations for how the zonular fibres alter lens shape^9,11,59^. The *in-vivo* behaviour of zonular fibres are not fully understood even though *in-vivo* imaging of zonules^23^ and the ciliary muscle^24^ have been reported. MRI images can display the whole lens shape but the images render the zonule hardly visible^41^. *In-vitro* studies that had stretched intact lenses cannot be certain that such stretching is an exact simulation of what happens in the eye^14,60–62^. By measuring the radius of curvature and calculating the central optical power of thirty *post-mortem* human lenses, Schachar concluded that the *post-mortem* lens, free of zonular tension, is actually in an unaccommodated state^63^, contradicting the conventional thinking that supports Helmholtz^9^, that *post-mortem* lenses are in an accommodated state. According to Schachar^11,59^, the anterior and posterior zonular fibres should be relaxed while the equatorial zonular fibre remains taut during accommodation. The greater change in the anterior and posterior zonule compared with the equatorial zonule with unaccommodation is not inconsistent with this. The results in Fig. 3b,c and d demonstrate an inflection point where radius of curvature increases while COP decreases for the first stretching step, similar to the response reported in a previous modelling study^64^.

It should be noted that whilst both Fisher^16^ and Wilde *et al*.^17^ used centrifugal force to calculate material properties, the former applied spinning forces to lenses that were capsulated^16^ whilst the latter used decapsulated samples^17^. The spinning lens test conducted by Fisher^16^ was the seminal study conducted on the elasticity of human lenses, but it was subsequently noted by Burd *et al*.^65^ that this work relied on simplifying assumptions that were (i) geometric, including treating the lens as a spheroid, assuming a similar radius for the nucleus as for the semi-minor axis of the lens (ii) mathematical: treating the lens as a stack of spinning discs and the cortico-nuclear boundary as partially adhered and (iii) mechanical made of isotropic, homogenous and linearly elastic material and with a negligible effect of the lens capsule^16^. This could have resulted in potential systematic errors notably the finding that the cortex is stiffer than the nucleus^16^. This notwithstanding, the current study sought to compare models from studies that used a similar technique: the early work of Fisher^16^ with the more recent findings of Wilde *et al*.^17^, who took into account assumptions that may have led to errors. Indentation measurements^34^ or those using a probe^35^ require sectioning or penetration of the lens that would induce alterations in the material properties of the lens that the spinning method avoids. The mechanical properties measured in studies that distinguish between the nucleus and the cortex dependent on assumptions about the nuclear shape, reports of which vary^16,17,34,35^. A limitation of the present study is that it uses nuclear shape data from optical images^42^ that are not the same as those used by Fisher^16^ and Wilde *et al*.^17^. Ideally material and shape parameters from the same eye need to be considered in future models that will also take account of individual variations and age-related differences.

The properties of biological materials depend on the constituent components and the interaction between these components^66^. The major constituents of the lens are crystallin proteins and water^67^ which vary in concentration across the lens and in interactions with water and this alters with age as more bound water becomes free^68^. Whilst there is also a change in lenticular material properties, how changes in crystallins and their interactions with water alter elastic moduli is not known. Such findings would need a combination of carefully controlled experimental studies that consider how variations in crystallin concentrations affect the moduli, as well as *in-vivo* measurements that can directly determine material properties in localised regions of the lens. Advanced techniques to make such measurements and account for the dynamic nature of a living lens would greatly advance knowledge in this field. It is notable that the concentration of proteins has been linked directly and linearly to the refractive index^69^ and the refractive index gradients are very similar to gradients of moduli^37^.

From the optimal p-values, indicating goodness of fits of lens models to *in-vivo* measurements, as well as from the plots of accommodative amplitudes, it can be seen that the equatorial zonular angles do not have a significant influence on lens shape change (Supplementary figures 1–4). A previous modelling study suggested that both theories could find support depending on the lens shapes, materials and applied forces used^70^. It is possible that the two accommodative theories are not completely contradictory. Models must be carefully constructed if they are to provide correct insights into accommodation and this study demonstrates that physiologically plausible models which compare to *in-vivo* measurements^40^ can respond in different ways to simulated stretching.

The present study describes a modelling approach that considers the effect of material properties, zonular angles and capsular thickness on changes in lens shape that can produce models that are comparable to a living lens^40^. The assumptions used in the modelling, largely dictated by the limitation on computational resources and simulation times, resulted in simplification of the biology. The number of zonular fibres attached to the lens capsule in the eye covers a far greater range of locations and angles than the three groups modelled in this work. The anterior hyaloid membrane, attached to the posterior lens capsule through Wieger’s ligament^71^, was not incorporated into current models. Recent evidence showing that the posterior zonule is anchored in the hyaloid membrane instead of attaching directly to the posterior lens capsule^72,73^, should be considered in future modelling studies. It is acknowledged that the lens is not an isolated component but an optical element in the eye. Ultimately whole eye models are needed for a range of applications from gaining a better understanding of ocular function to design of implants and prediction of changes post-surgically. To be physiologically relevant, lens models need to be constructed based on comparison to data from live eyes. Simulated changes in lens shape must adhere to changes in shape that occur when the living lens accommodates. Realistic models of the lens can subsequently be incorporated in the construction of whole eye models that take into account the optics of the eye and can ultimately advance the design of personalised implants

## Conclusions

Lens models using geometries and material properties from previous *in-vitro* studies were compared to *in-vivo* results to select zonular configurations that would be physiologically relevant. The equatorial zonule does not appear to play as significant a role on lens shape change as do the anterior and posterior zonular fibres. Changing zonular angles with age may account for the accommodative loss as zonular angle triplets that provide results more closely aligned to *in-vivo* data give lower accommodative amplitudes than those which produce highest optical performance. Capsular thickness should be taken into account in modelling as it may have an effect on the optical performance of the lens.

## Methods

### Geometries and materials

The basic design parameters used in the models are: (i) Age: shape profiles from measurements on 16-year-old and 35-year-old lenses^42^ were fitted in SolidWorks ver.2017 using two splines for the nuclear contours and the outer lens shape respectively. The dimensions of the two lenses are given in Fig. 4a and c and the parameters defining the splines in SolidWorks are listed in Table 2. (ii) Material properties: two different sets of values were used from the measurements of Fisher^16^ and Wilde *et al*.^17^ with the corresponding values listed in Table 2; (iii) Capsular thickness: two different cases were studied: one with uniform thickness^19^: 13 µm for the 16-year-old lens and 15 µm for the 35-year-old lens, and one a varying thickness in accordance with comparable age-related values from Barraquer *et al*.^39^: the thickness profiles of a 12-year-old lens and a 33-year-old lens^39^ were used for the 16-year-old lens model and the 35-year-old lens model respectively. Additional uniform capsular thickness models were simulated with values measured from both anterior and posterior capsules by Krag and Andreasson^38^ with the values listed in Table 1 to investigate the influence of capsular thickness on the optical performance of lens models. The combination of the aforementioned design parameters yielded a total of sixteen different models which were developed in ANSYS Mechanical version 18.1, using the intrinsic APDL programming language. Each model developed in the present study consists of six parts: (i) nucleus, (ii) cortex, (iii) capsule, (iv) anterior zonular fibre section, (v) equatorial zonular fibre section and (vi) posterior zonular fibre section.

The zonular fibres were anchored on the lens capsule using a master-slave approach to maintain a smooth lens curvature with deformation and avoid kinks of numerical origin. The master nodes were put at points A_1_, P_2_ and A_2_, as shown in Fig. 4a and c, while the slave nodes were spread around the master nodes, as indicated with purple triangles in Fig. 4b and d. In this way, the degrees of freedom of the slave nodes were appropriately coupled to the degrees of freedom of the master nodes so that former slave nodes could follow the movement of each zonular anchorage point. The dimension of the central zonular-free area^20,21,74^ for each model was shown in Fig. 4b and d.

With respect to the angles of the zonular fibre sections, the domain for ${\theta }_{a}$, was between 10° and 30° towards the posterior of the eye (represented as [10°, 30°]), the domain for ${\theta }_{e}$, was [−14°, 14°] (the negative sign denoting the posterior direction and the positive sign denoting the anterior direction for ${\theta }_{e}$ only) and the domain for ${\theta }_{p}$, was [24°, 44°] towards the anterior of the eye (Fig. 4a,c). In order to carry out a systematic investigation within the parameter space defined by these domains, a step size of 2° was used for each zonular angle, giving a total of 1815 different zonular angle triplets per model.

All constituents of the models were treated as isotropic, linear elastic and homogenous. Young’s moduli were 5.87 MPa and 4.90 MPa respectively for the capsules^19^ of 16 and 35 aged lenses respectively and 0.35 MPa for the zonular fibres^75,76^. Poisson’s ratio for both the capsule and the zonular fibres was 0.47^19,77^. Each zonular fibre had a length of 1.5mm^60^ and a thickness of 0.05mm^78^.

### Finite Element Model development

The lens nucleus, cortex and capsule were considered as fully axisymmetric bodies and meshed with appropriate finite elements. The nucleus and cortex were meshed using higher-order 8-node plane elements with the axisymmetric option enabled (ANSYS element type: PLANE 183), this element has a quadratic displacement behaviour and is well suited for irregular meshes. The capsule was considered as a thin-walled membrane structure and was meshed with 3-node axisymmetric shell elements (ANSYS element type: SHELL 209); this element is well suited for linear, large rotation, and/or large strain nonlinear applications. Finally, as an axisymmetric approach was selected, each set of zonular fibres was considered as a continuous sheet^22^ and therefore meshed with 2-node shell elements with only membrane stiffness, with the axisymmetric option enabled and the torsional capability disabled (ANSYS element type: SHELL 208). The total number of elements was 1131 and the total number of nodes was 5500 for all models. Nonlinear geometrical analyses were conducted for all models.

### Applied procedure

**Figure 5:**
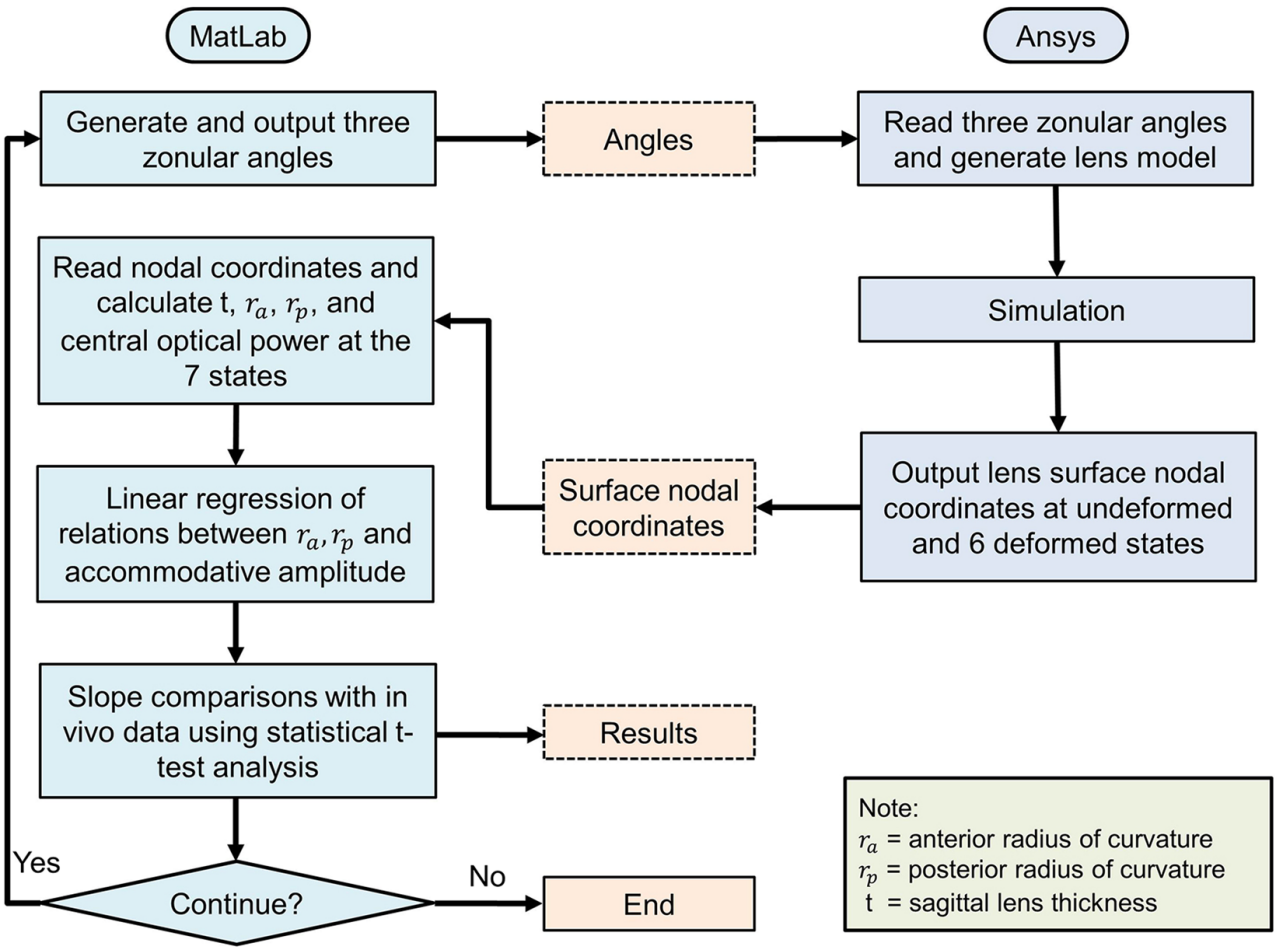
Flow diagram indicating methodologic steps applied in the modelling procedure.

The procedure applied is shown in Fig. 5. In total, two codes were developed, one in MatLab (Ver.2015b) and one in ANSYS/Mechanical APDL (Ver.18). The MatLab code was used to (a) generate zonular angle triplets composed of angles for the anterior, equatorial and posterior zonular sections, (b) call ANSYS, in batch mode, as an external solver for the respective Finite Element (FE) analysis, (c) retrieve results from the FE analysis, (d) calculate the necessary quantities for the comparison between the *in-vivo* lens^40^ and the obtained numerical results and (e) conduct a statistical t-test between the *in-vivo* ^40^ and the obtained numerical results. The ANSYS/APDL code was used to (a) read zonular angle triplets generated with the MatLab code, (b) build the respective axisymmetric CAD model of the human lens based on a predefined input file containing all the information for the lens geometry and all material properties, (c) setup the FE model (i.e. define finite element types, material properties, boundary conditions), (d) run the simulation and (e) retrieve results from the FEA simulations. For each simulation, the lens was supported to avoid in-plane rigid body motions. Furthermore, two sets of displacements with predefined magnitude and direction were imposed on the endpoint of each zonular fibre anchored to the ciliary body: (1) 0.6 mm displacements applied to all three sets of zonular fibres; (2) 0.5 mm applied to the equatorial zonular fibre and 0.6 mm applied to the anterior and posterior zonular fibres. The selection of the magnitude of displacements accords with an MRI study^41^, which showed a maximal change in ciliary ring diameter between 1 mm to 1.2 mm. The aforementioned total displacements were introduced in six equal increments and applied to the lens model sequentially.

**Figure 6:**
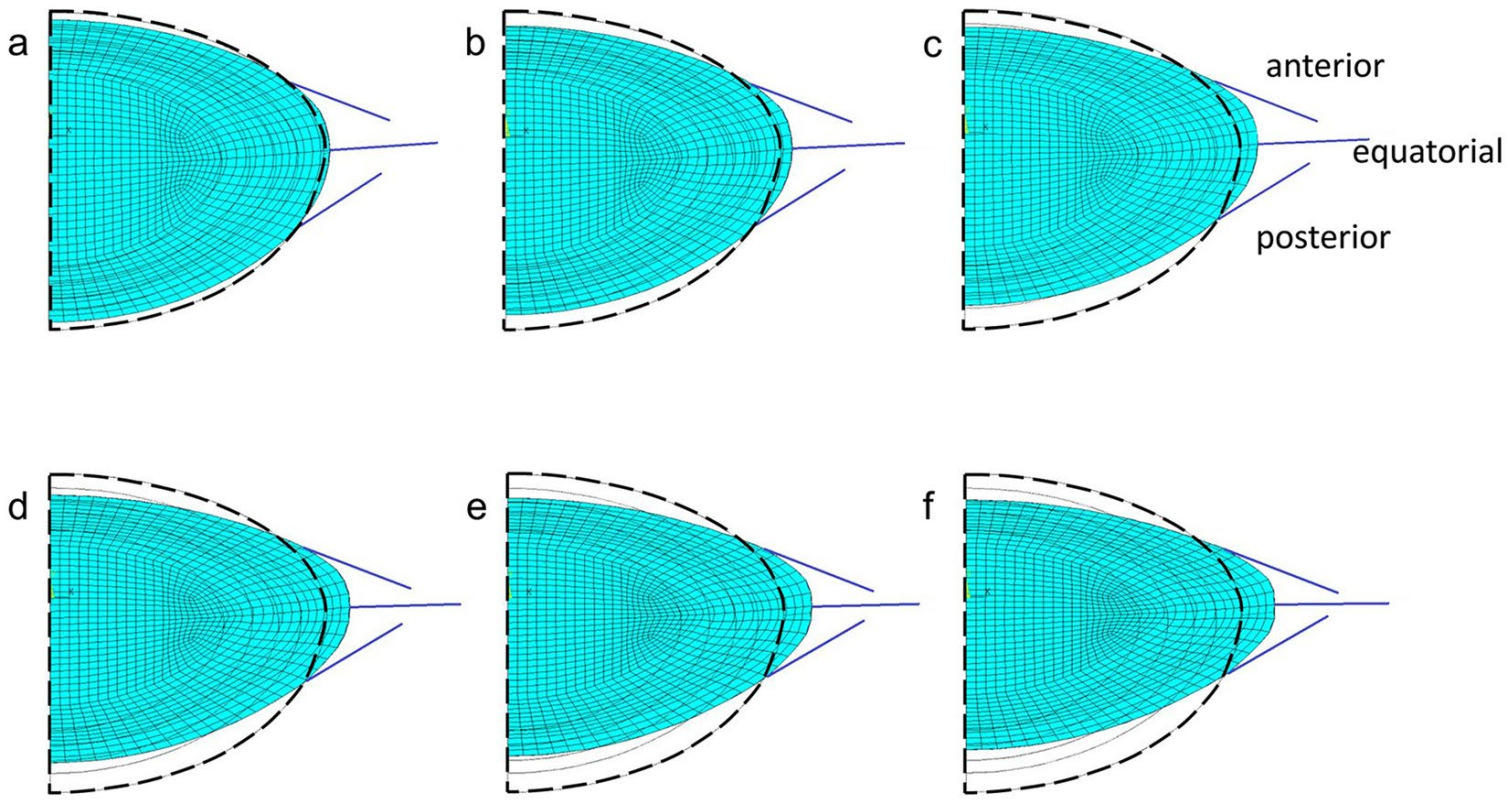
A representation of Finite Element lens models created showing a 16-year-old lens model with three parts of the zonule at angles of 20 degrees anterior, 4 degrees equatorial and 34 degrees posterior for six stages of deformation. The dashed contour indicates the non-deformed shape.

Figure 6 shows a representative case, for the triplet (${\theta }_{a}$, ${\theta }_{e}$, ${\theta }_{p}$) = (20°, 4°, 34°), with the deformed shapes from a set of six increments. From completion of each increment, four quantities were calculated: (i) the radius of curvature of the lens anterior surface (${r}_{a}$), (ii) the radius of curvature of the lens posterior surface (${r}_{p}$)^79^, (iii) the sagittal thickness (*t*) and (iv) the Central Optical Power (COP) of the lens. These calculations were based on nodal coordinates taken from the undeformed and deformed shapes of the lens anterior and posterior surfaces and within the central paraxial 3 mm zone of the lens. The calculation of COP was based on equation (1)^80^.

1 $$COP=\frac{{n}_{1}-{n}_{a}}{{r}_{a}}+\frac{{n}_{1}-{n}_{a}}{{r}_{p}}-\frac{t{({n}_{1}-{n}_{a})}^{2}}{{r}_{a}{r}_{p}{n}_{1}}$$

where ${n}_{a}$=1.336 is the refractive index of the aqueous humour and ${n}_{1}$=1.42 is the equivalent refractive index of the lens^80^.

### Statistical analysis

To simulate the physiological situation, the maximal deformed state (seen in Fig. 6f) is defined as the reference state which is the accommodative demand for an eye focussed on distant objects and difference in the COP for progressively less deformation and the reference state were determined. The standard statistical t-test for comparing two slopes of independent samples^81^ was applied to compare changes in radii of curvature, for both the anterior and posterior lens surfaces, plotted against the change in COP (Fig. 1) from the simulations with those from the *in-vivo* lens^40^, which produced two p-values per model (i.e. one p-value for the curvature of the anterior and one p-value for the curvature of the posterior lens surface, respectively). A significance level of 0.05 was used to select triplets (${\theta }_{a}$, ${\theta }_{e}$, ${\theta }_{p}$) that compared with the *in-vivo* lens. The null hypothesis of the statistical test is that there is no significant difference between the simulated slope and the *in-vivo* measured slope. Triplets that resulted in a p-value lower than 0.05 thereby rejecting the null hypothesis were not shown in the p-value contours. When both anterior and posterior p-values were greater than 0.05, the corresponding triplet was considered as an acceptable combination of zonular angles and statistically close to the *in-vivo* data^40^. Otherwise this triplet was rejected as non-physiological. The aforementioned procedure was repeated for all models and for an exhaustive search of 1815 zonular angle triplet combinations per model.

## Electronic supplementary material

Supplementary information
