# Supplementary material for: The importance of parameter choice in modelling dynamics of the eye lens
Source: Sci Rep. 2017 Nov 30;7:16688. doi: 10.1038/s41598-017-16854-9 (PMC5709469; doi:10.1038/s41598-017-16854-9)
Supplement: Supplementary file 1 — Supplementary information [file 41598_2017_16854_MOESM1_ESM.pdf]

The importance of parameter choice in modelling dynamics of the eye lens

Kehao Wang[1], Demetrios T. Venetsanos[1], Jian Wang[1], Andy T.Augousti[1], *Barbara K. Pierscionek[2]

1. Faculty of Science Engineering and Computing Penrhyn Road

   Kingston-upon-Thames, KT1 2EE UK

2. College of Science and Technology Nottingham Trent University, DH Lawrence Building, Clifton Campus Clifton Lane Nottingham NG11 8NS, UK

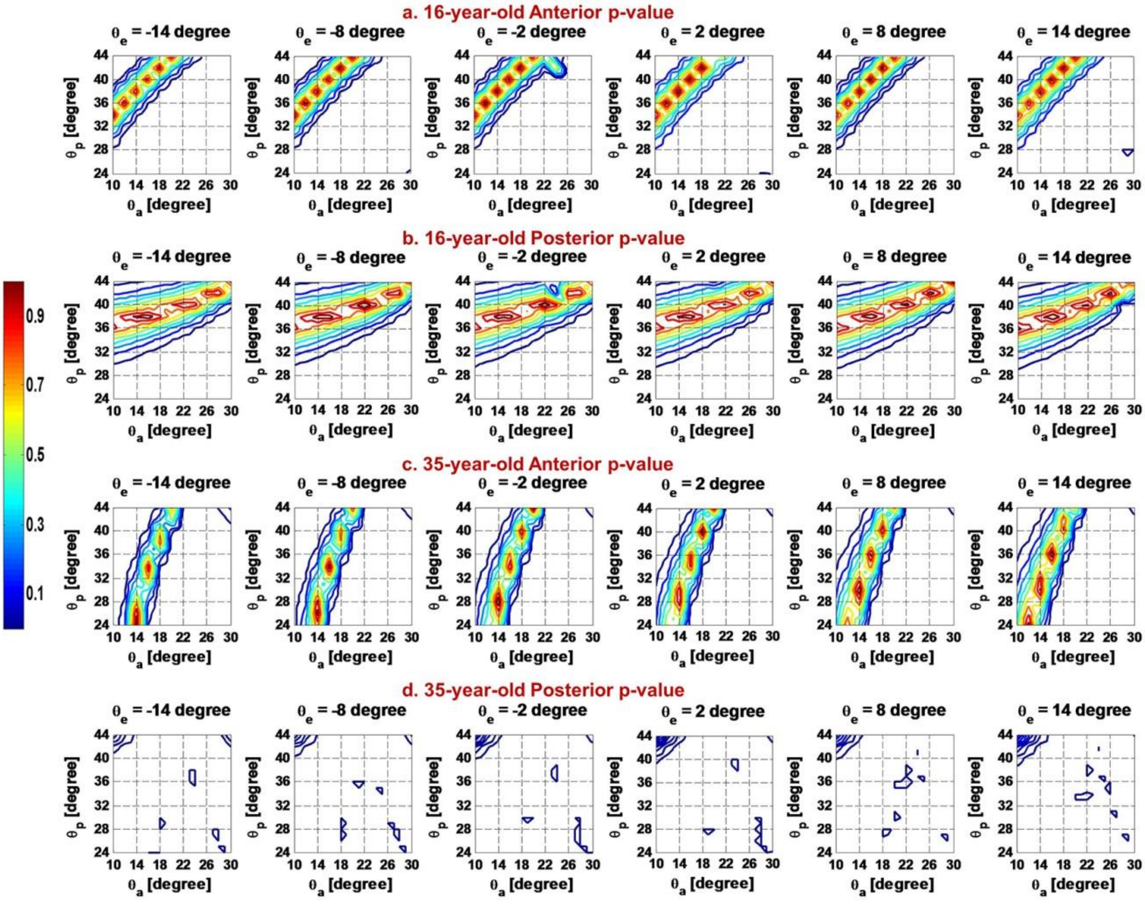

Suppl Fig 1. The p-values for different combinations of zonular triplets shown as contours for (a) the anterior lens surface and (b) the posterior lens surface of the 16-year-old lens model , (c) the anterior lens surface and (d) the posterior lens surface of the 35-year-old lens model using material properties of Fisher[16] and for a uniform capsular thickness[19], with 0.5mm applied to the equatorial zonule, 0.6mm applied to the anterior and posterior zonules.

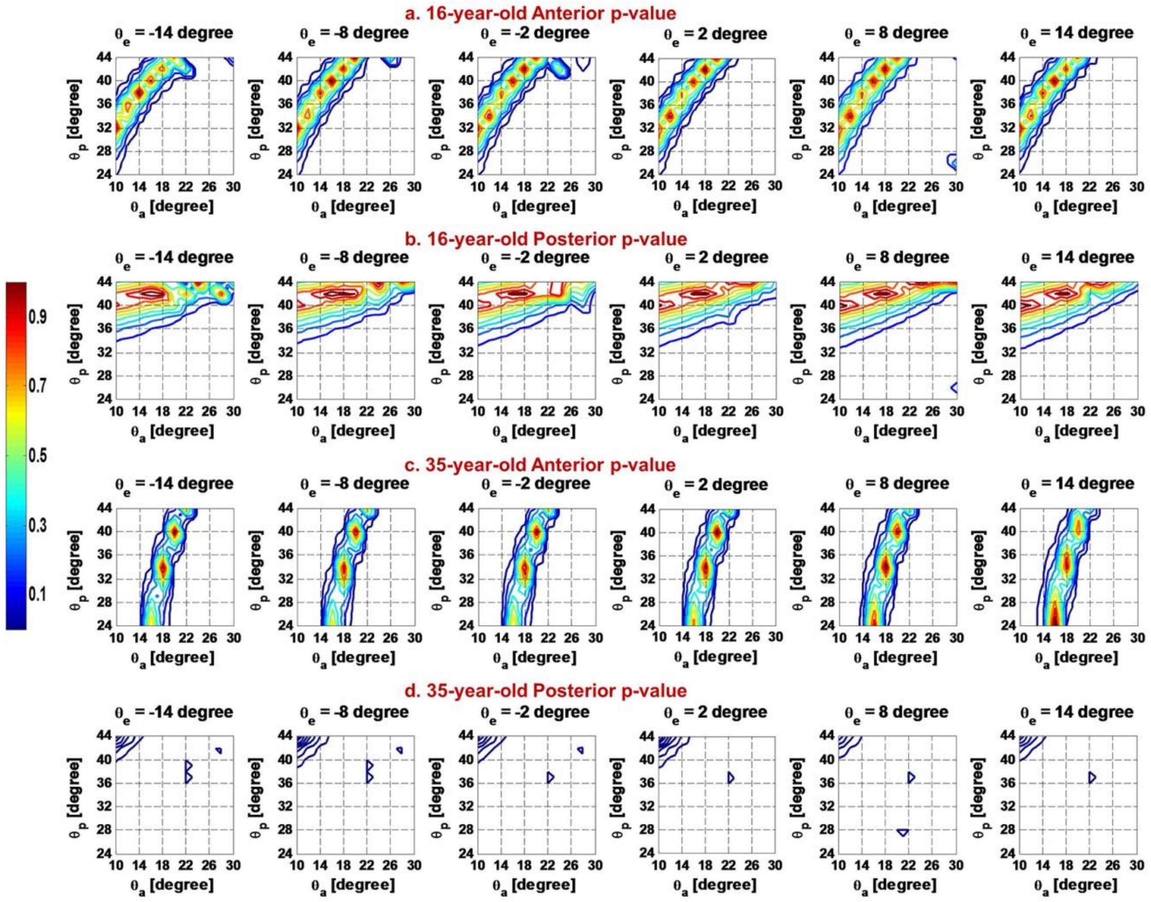

Suppl Fig 2. The p-values for different combinations of zonular triplets shown as contours for (a) the anterior lens surface and (b) the posterior lens surface of the 16-year-old lens model , (c) the anterior lens surface and (d) the posterior lens surface of the 35-year-old lens model using material properties of Fisher[16] and for a varying capsular thickness[39], with 0.5mm applied to the equatorial zonule, 0.6mm applied to the anterior and posterior zonules.

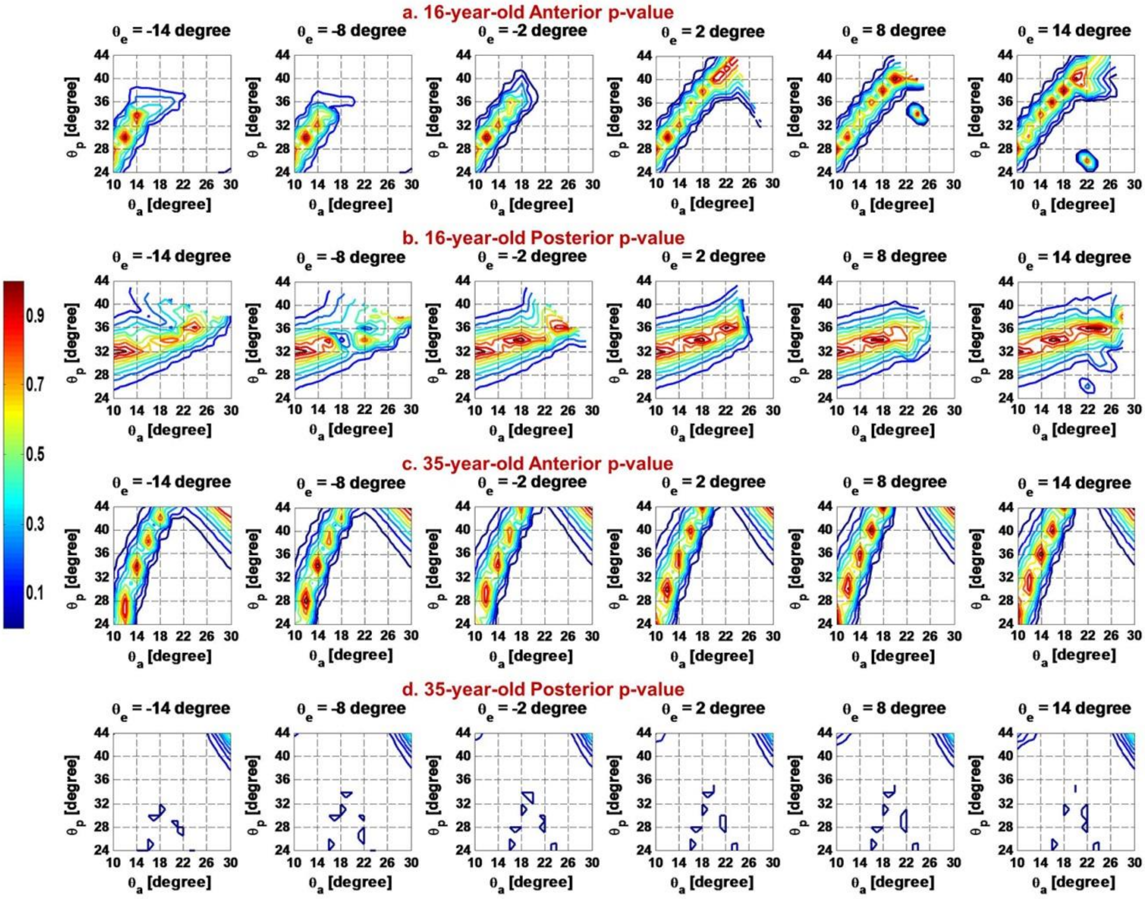

Suppl Fig 3. The p-values for different combinations of zonular triplets shown as contours for (a) anterior lens surface and (b) posterior lens surface of the 16-year-old lens model, (c) the anterior lens surface and (d) the posterior lens surface of the 35-year-old lens model using material properties of Wilde *et al.*[17] and for a uniform capsular thickness[19], with 0.5mm applied to the equatorial zonule, 0.6mm applied to the anterior and posterior zonules.

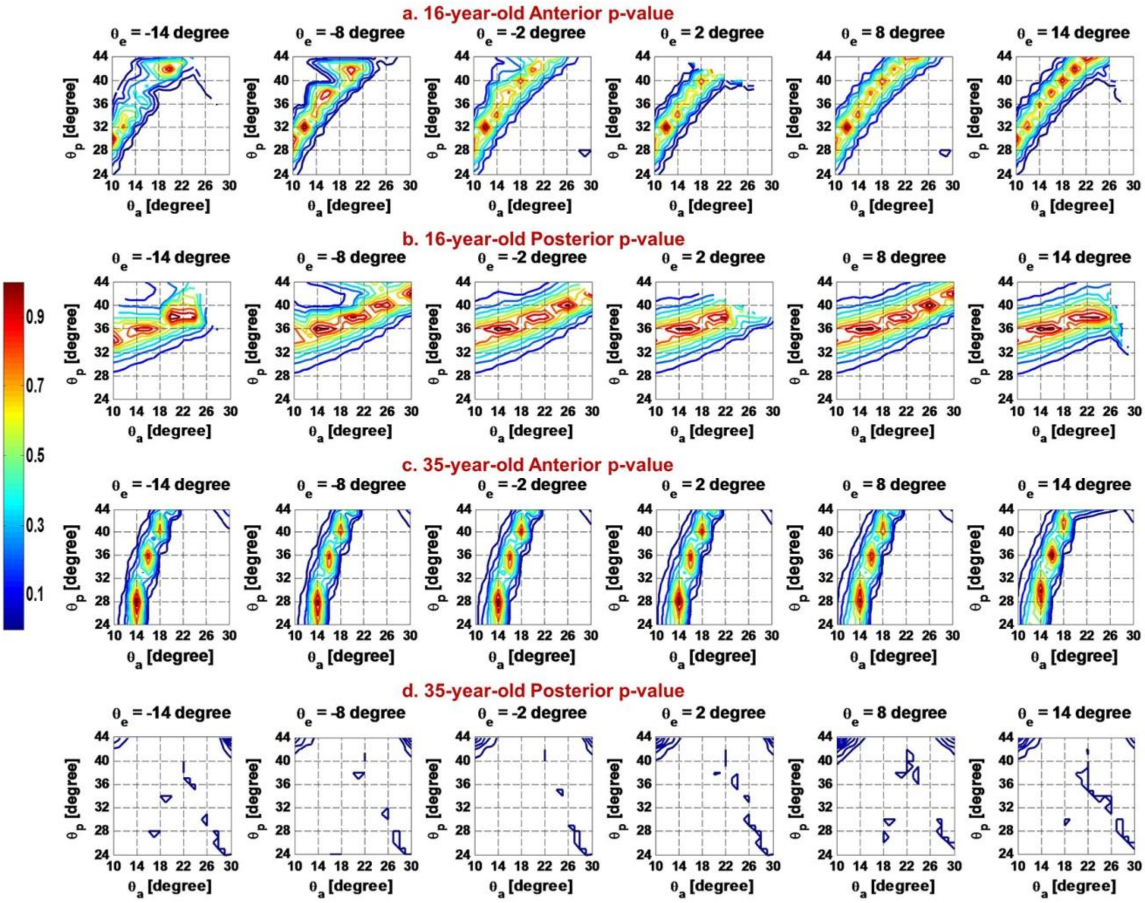

Suppl Fig 4. The p-values for different combinations of zonular triplets shown as contours for (a) anterior lens surface and (b) posterior lens surface of the 16-year-old lens model, (c) the anterior lens surface and (d) the posterior lens surface of the 35-year-old lens model using material properties of Wilde *et al.*[17] and for a varying capsular thickness[39], with 0.5mm applied to the equatorial zonule, 0.6mm applied to the anterior and posterior zonules.
